# Associations between selected genetic variants and lipid profile variability in response to statins in Alzheimer’s disease: a prospective observational study

**DOI:** 10.1590/1516-3180.2024.0160.27112024

**Published:** 2025-08-15

**Authors:** Fabricio Ferreira de Oliveira, Sandro Soares de Almeida, Elizabeth Suchi Chen, Paulo Henrique Ferreira Bertolucci, Marilia Cardoso Smith

**Affiliations:** I MD, MSc, PhD, FAAN. Departamento de Neurologia e Neurocirurgia, Escola Paulista de Medicina, Universidade Federal de São Paulo (Unifesp), São Paulo (SP), Brazil.; II MSc, PhD. Departamento de Biofísica, Escola Paulista de Medicina, Universidade Federal de São Paulo (Unifesp), São Paulo (SP), Brazil.; III MSc, PhD. Professor, Departamento de Morfologia e Genética, Escola Paulista de Medicina, Universidade Federal de São Paulo (Unifesp), São Paulo (SP), Brazil.; IV MD, MSc, PhD. Professor, Departamento de Neurologia e Neurocirurgia, Escola Paulista de Medicina, Universidade Federal de São Paulo (Unifesp), São Paulo (SP), Brazil.; V MSc, PhD. Professor, Departamento de Morfologia e Genética, Escola Paulista de Medicina, Universidade Federal de São Paulo (Unifesp), São Paulo (SP), Brazil.

**Keywords:** Pharmacogenetics, Receptors, lipoprotein, Renin-angiotensin system, Apolipoproteins E, Hydroxymethylglutaryl-CoA reductase inhibitors, Alzheimer disease, Dementia, Cholesterol, Drug therapy

## Abstract

**BACKGROUND:**

Lipid profiles are largely determined by genetic variants, and lipid metabolism plays a crucial role in Alzheimer’s disease.

**OBJECTIVE:**

To investigate whether lipid profile variability in response to diverse statins could be affected by cholesterol metabolism-related genetic variants in Alzheimer’s disease..

**DESIGN AND SETTING:**

This prospective observational pharmacogenetic study was conducted at the Universidade Federal de São Paulo (Unifesp), Brazil.

**METHODS:**

Consecutive outpatients were prospectively followed for lipid profile variations over one year, estimated by the associations between statin therapy and the following variants: rs2695121 (NR1H2), rs3846662 (HMGCR), rs11669576 (LDLR8), rs5930 (LDLR10), rs5882 and rs708272 (CETP), rs7412 and rs429358 (APOE), and ACE insertion/deletion polymorphism.

**RESULTS:**

All polymorphisms in the 189 patients were in Hardy-Weinberg equilibrium. Statins resulted in lower total cholesterol and LDL cholesterol levels, whereas the effects on HDL cholesterol varied according to the statin used. Atorvastatin resulted in lower triglyceride level variations than simvastatin. APOE-ε4 carriers showed a better response to atorvastatin in elevating HDL-cholesterol than APOE-ε4 non-carriers. Carriers of the ACE insertion allele had cumulatively lower total cholesterol and LDL-cholesterol levels, regardless of statin therapy, but lower triglyceride levels when using atorvastatin. Carriers of rs11669576-G had lower total cholesterol and LDL-cholesterol levels when using simvastatin, and lower total cholesterol and triglycerides when using atorvastatin. Concerning CETP haplotypes, carriers of rs5882-A and rs708272-A benefitted the most from statins, which lowered total cholesterol and increased HDL-cholesterol levels, and from atorvastatin lowering triglycerides; however, the effects of atorvastatin lowering total cholesterol and LDL-cholesterol were more pronounced for carriers of rs5882-GG/rs708272-GG.

**CONCLUSION:**

Lipid profile variations may be pharmacogenetically mediated in Alzheimer’s disease, thus, confirming their high heritability.

## INTRODUCTION

Cerebrovascular risk factors may be more important in the pathogenesis of Alzheimer’s ­disease compared to other primary neurodegenerative diseases;^1^ however, an intimate relationship exists between lipid metabolism and Alzheimer’s disease. Midlife hypercholesterolemia in combination with other vascular risk factors causes earlier onset of late-onset Alzheimer’s disease dementia.^2^ However, such combinations may be protective against cognitive decline in patients with established dementia, possibly due to enhanced late life cerebral perfusion.^3^ For instance, carriers of the ε4 allele of the apolipoprotein E (APOE) gene with a high 10-year coronary heart disease risk (dependent upon vascular risk factors such as higher total cholesterol and lower HDL-cholesterol), may have slower cognitive decline in the mild dementia stage.^4^

Genetic variants are responsible for approximately 30% of the variability in fasting blood cholesterol concentrations.^5^ For instance, APOE in chromosome 19 is the most important gene that affects the incidence and the age at onset of late-onset Alzheimer’s disease dementia,^2^ as it encodes a key extracellular lipid transport protein produced mostly by hepatocytes, which is responsible for clearance of ­triglyceride-rich lipoproteins.^6^ The Alu repeat insertion/deletion polymorphism, within intron 16 of the angiotensin-converting enzyme gene (ACE) in chromosome 17, is associated with obesity^7^ and higher blood pressure when boosting serum levels of the angiotensin-converting enzyme in the presence of the deletion allele.^8^ This polymorphism could potentially affect white matter myelin loss in Alzheimer’s disease as well as behavioral features;^9^ however, ­limited studies have shown associations between this polymorphism and the dementia syndrome.^10^ The 3-hydroxy-3-methylglutaryl CoA reductase gene (HMGCR) in chromosome 5 is the mechanistic gene that encodes the statin-binding domain of the enzyme 3-hydroxy-3-methylglutaryl CoA reductase, which is involved in the regulation of the intracellular biosynthesis of cholesterol.^11^ Furthermore, the intronic A allele of rs3846662 (HMGCR) is associated with lower LDL-cholesterol levels and reduced response to statin therapy.^12^ Single nucleotide polymorphisms rs11669576 (LDLR8) and rs5930 (LDLR10) are among the prime genetic variants of the epidermal growth factor precursor homology domain of LDLR (the low-density lipoprotein cholesterol receptor gene) in chromosome 19 to be associated with dysfunctional cholesterol metabolism.^13^ NR1H2 in chromosome 19 encodes the liver X receptor β (LXR-β) isoform. When underexpressed, NR1H2 increases cellular cholesterol ­levels and amyloidogenesis by downregulating the apolipoprotein E.^14^ Specifically, the T allele of rs2695121 (NR1H2) may affect the risk of Alzheimer’s disease and produce higher behavioral burden.^15^ The cholesteryl ester transfer protein gene (CETP) in chromosome 16 encodes the hydrophobic cholesteryl ester transfer protein,^11^ which is involved in reverse cholesterol transport (from peripheral tissues to the liver) and promotes the uptake of cholesterol in the liver by mediating the transfer of cholesteryl esters from high-density lipoproteins to apolipoprotein B-containing lipoproteins in exchange for triglycerides.^6^ The protective CETP variants of rs5882 and rs708272 lead to lower serum levels of the cholesteryl ester transfer protein and, consequently, healthier lipid profiles.^15^

Statins are the most commonly prescribed lipid-lowering agents,^6^ although they can be underused in older people because of their side effects.^16^ They lower plasma cholesterol concentrations by competitively inhibiting the enzyme 3-hydroxy-3-methylglutaryl-CoA reductase, providing benefits to patients with Alzheimer’s disease because of their lipid-lowering effects by blocking cholesterol biosynthesis and upregulating the low-density lipoprotein receptor, and because of their antioxidant, antithrombotic, antiamyloidogenic, and vasodilatory properties.^17^

## OBJECTIVE

In this longitudinal pharmacogenetic study, we aimed to determine whether variability in lipid profiles in response to diverse statins could be affected by selected genetic variants related to cholesterol metabolism in a cohort of outpatients with Alzheimer’s disease dementia.

## METHODS

### Participants and clinical assessment

This prospective observational study recruited a convenience sample of consecutive outpatients with Alzheimer’s disease dementia, based on the National Institute on Aging-Alzheimer’s Association criteria^18^, from the Behavioral Neurology Section of our university hospital between October 2010 and May 2014. After confirmation of the neurological diagnosis, all patients from this uncontrolled cohort were followed up for one year and had at least three consultations in which they were assessed for proxy reports regarding age, alcohol use, smoking, and lipid-lowering therapy with a statin; sex and body mass index were also assessed. Participation of each patient was concluded when the one year follow-up was completed. Only the first and last evaluations were considered for statistical analysis.

Diagnoses of dyslipidemias^19^ and diabetes mellitus^20^ were based on blood tests, whereas that of arterial hypertension was based on the JNC 7^21^ report. Statin therapy was monitored year-long. Dyslipidemia was managed according to specific guidelines.^19^ Essentially, total cholesterol (desirable < 200 mg/dl), LDL-cholesterol, HDL-cholesterol, VLDL-cholesterol, and triglycerides target levels were determined based on the presence or absence of symptomatic ischemic heart disease, coronary heart disease risk equivalents (diabetes mellitus and clinical manifestations of non-coronary forms of atherosclerotic disease), and cardiovascular risk factors. Statins were introduced only if lifestyle recommendations were unsuccessful after three months, or unless the 10-year estimated coronary heart disease risk was > 10%. Lifestyle recommendations, including body weight control, regular physical activity, dietary therapy, and smoking cessation, were employed for all patients.^22^ Statin therapy was discontinued in case of side effects. All efforts were directed towards reducing cholesterol and triglyceride levels to their target levels.

### Genotyping

Genomic DNA was extracted from whole blood using a standard salting-out procedure. Subsequently, the following genotypes were determined by real-time Polymerase Chain Reactions: rs3846662 (HMGCR), rs2695121 (NR1H2), rs11669576 (LDLR8), rs5930 (LDLR10), rs5882 and rs708272 (CETP), and rs7412 and rs429358 (APOE). The use of TaqMan^®^ SNP Genotyping Assays on the Applied Biosystems 7500 Fast Real-Time PCR System (Applied Biosystems, Foster City, California, United States) was in accordance with standard protocols. In addition, the ACE Alu insertion/deletion (I/D) polymorphism was determined using conventional polymerase chain reactions.^23^ Genotyping procedures were performed after clinical data were collected from all patients.

### Outcome measures

The primary outcome measure was variation in the levels of triglycerides, total cholesterol, LDL-cholesterol, and HDL-cholesterol according to the following independent variables: APOE haplotypes and genotypes of ACE I/D, HMGCR, NR1H2, LDLR or CETP, and lipid-lowering therapy with a statin. The secondary outcome was variation in the levels of triglycerides, total cholesterol, LDL-cholesterol, and HDL-cholesterol according to different statins. Haplotypes of LDLR and CETP were also considered in the analyses.

### Statistical analyses

The Hardy-Weinberg equilibrium was estimated using the chi-square test. Lipid profile variables are expressed as absolute values and treated as continuous measures. A paired Student’s t-test was used to assess variations in body mass index and lipid profile variables (considering baseline and final values after one year). Variations in triglycerides, total cholesterol, LDL cholesterol, and HDL-cholesterol at one year were then logarithmically transformed to meet the normality assumptions for a general linear model that was employed to evaluate associations between variations in these lipid profile variables with every genetic variant and statin therapy and adjusted for the following covariates: sex, baseline age, and body mass index variations in one year. Levels of significance from the general linear model were corrected for false discovery rates according to the Benjamini-Hochberg procedure to minimize the occurrence of type I errors. However, due to the preliminary nature of this study, the final results were presented with and without correction. Univariate analyses revealed the effects of statin therapy on lipid profile variations regardless of genetic variants, while multivariate analyses showed the results of interactions between genetic variants and the use or non-use of a statin on lipid profile variations. Concerning APOE haplotypes, the number of APOE-ε4 alleles was considered for the analysis rather than genotypes. Statistical significance was set at P < 0.05.

The dataset that was used for all statistics of this article is freely available for download at Mendeley Data (https://doi.org/10.17632/6n7z6hytrj.1).

### Ethical considerations

All procedures in this study 1067/10 (CAAE 0540.0.174.000-10) were approved by the Ethics Committee of our university hospital on August 20, 2010 and were performed in accordance with the recommendations of the Declaration of Helsinki on Biomedical Research involving Human Participants. All invited patients and their legal representatives provided consent via a signed Informed Consent Form before assessment.

## RESULTS

A total of 217 consecutive outpatients with late-onset Alzheimer’s disease dementia were included in this study; 14 (6.5%) died, 7 (3.2%) were lost during follow-up, and 7 (3.2%) were excluded because of incomplete clinical data, resulting in a final cohort of 189 patients.

Table 1 presents the demographic, anthropometric, and clinical characteristics of the study population. Almost two-thirds of the patients were women, and exhibited a high prevalence of vascular risk factors. The use of lipid-lowering therapy was highly prevalent, with most patients using the lipophilic statins simvastatin or atorvastatin. The mean body mass index, total cholesterol, and LDL cholesterol were significantly lower after one year of treatment with statins, however, no significant differences were observed in the mean values of triglycerides, VLDL cholesterol, or HDL cholesterol.

**Table 1.** Demographics and Clinical Features

Assessed Variables, n = 189		n (%)	Mean ± SD	P*
Sex	Women	124 (65.6%)	-	-
	Men	65 (34.4%)	-	-
Estimated age at dementia onset		-	73.24 ± 6.5 years-old	-
Baseline age		-	78.24 ± 5.9 years	-
Body mass index	Baseline values	-	25.61 ± 4.3 kg/m²	P < **0.0001**
	Final values	-	24.96 ± 4.6 kg/m²	
	Variation	-	−0.65 ± 2.2 kg/m²	-
Arterial hypertension		152 (80.4%)	-	-
Diabetes mellitus		52 (27.5%)	-	-
Dyslipidemia		141 (74.6%)	-	-
Lifetime alcohol use		45 (23.8%)	62.24 ± 72.6 L/year^a^	-
Current alcohol use		2 (1.1%)	102.50 ± 109.6 L/year^a^	-
Lifetime smoking		65 (34.4%)	133.48 ± 154.2 packs/year^b^	-
Current smoking		7 (3.7%)	101.43 ± 62 packs/year^b^	-
Statin therapy	Simvastatin	124 (65.6%)	18.79 ± 9.4 mg/day^c^	-
	Atorvastatin	14 (7.4%)	28.57 ± 22.8 mg/day^c^	-
	Rosuvastatin	2 (1.1%)	10 ± 0 mg/day^c^	-
No lipid-lowering therapy		49 (25.9%)	-	-
Total cholesterol	Baseline values	-	197.96 ± 46.7 mg/dL	P < **0.0001**
	Final values	-	181.25 ± 38.2 mg/dL	
	Variation	-	−16.71 ± 36.8 mg/dL	-
LDL-cholesterol	Baseline values	-	118.02 ± 40.4 mg/dL	P < **0.0001**
	Final values	-	101.59 ± 29.5 mg/dL	
	Variation	-	−16.43 ± 33.1 mg/dL	-
HDL-cholesterol	Baseline values	-	53.08 ± 14.6 mg/dL	P = 0.843
	Final values	-	53.22 ± 15.2 mg/dL	
	Variation	-	0.14 ± 9.7 mg/dL	-
VLDL-cholesterol	Baseline values	-	26.85 ± 12.1 mg/dL	P = 0.612
	Final values	-	26.44 ± 12.7 mg/dL	
	Variation	-	−0.42 ± 11.3 mg/dL	-
Triglycerides	Baseline values	-	134.26 ± 60.7 mg/dL	P = 0.606
	Final values	-	132.14 ± 63.3 mg/dL	
	Variation	-	−2.12 ± 56.3 mg/dL	-

SD = standard deviation. * Paired Student’s t-test. ^a^ Mean l/year only for those with a history of alcohol use. ^b^ Mean packs/year only for those with a history of smoking. ^c^ Mean mg/day only for those who used the drug.

Table 2 shows the results of the genetic analyses. Although all variants were in Hardy-Weinberg equilibrium, all patients had the same rs3846662 (HMGCR) genotype (AA). Among them, 53.4% were APOE-ε4 carriers and 46.6% were APOE-ε4 non-carriers. All CETP haplotypes were represented in the sample, however, the low variability of genotypes of rs11669576 (LDLR8) led to the underrepresentation of some LDLR haplotypes.

**Table 2.** Genotypes and Haplotypes

Genetic Variants, n = 189			n (%)	P*
APOE haplotypes	APOE-ε4 carriers, n = 101	ε4/ε4	21 (11.1%)	-
		ε4/ε3	72 (38.1%)	-
		ε4/ε2	8 (4.2%)	-
	APOE-ε4 non-carriers, n = 88	ε3/ε3	81 (42.9%)	-
		ε3/ε2	7 (3.7%)	-
		ε2/ε2	0 (0%)	-
ACE Alu repeat insertion/deletion genotypes		I/I	50 (26.5%)	P = 0.345
		I/D	88 (46.6%)	
		D/D	51 (26.9%)	
HMGCR (intron 13): rs3846662 genotypes		AA	189 (100%)	P = 1
		AG	0 (0%)	
		GG	0 (0%)	
LDLR single nucleotide polymorphisms	exon 8: rs11669576 genotypes	AA	1 (0.5%)	P = 0.850
		AG	28 (14.8%)	
		GG	160 (84.7%)	
	exon 10: rs5930 genotypes	AA	23 (12.2%)	P = 0.749
		AG	83 (43.9%)	
		GG	83 (43.9%)	
LDLR haplotypes	rs11669576 AA/rs5930 AA		0 (0%)	-
	rs11669576 AA/rs5930 AG		0 (0%)	-
	rs11669576 AA/rs5930 GG		1 (0.5%)	-
	rs11669576 AG/rs5930 AA		0 (0%)	-
	rs11669576 AG/rs5930 AG		10 (5.3%)	-
	rs11669576 AG/rs5930 GG		18 (9.5%)	-
	rs11669576 GG/rs5930 AA		23 (12.2%)	-
	rs11669576 GG/rs5930 AG		73 (38.6%)	-
	rs11669576 GG/rs5930 GG		64 (33.9%)	-
NR1H2 (intron 2): rs2695121 genotypes		CC	72 (38.1%)	P = 0.954
		CT	89 (47.1%)	
		TT	28 (14.8%)	
CETP single nucleotide polymorphisms	rs5882 genotypes	AA	71 (37.6%)	P = 0.407
		AG	94 (49.7%)	
		GG	24 (12.7%)	
	rs708272 genotypes	AA	20 (10.6%)	P = 0.051
		AG	101 (53.4%)	
		GG	68 (36%)	
CETP haplotypes	rs5882 AA/rs708272 AA		3 (1.6%)	-
	rs5882 AA/rs708272 AG		34 (18%)	-
	rs5882 AA/rs708272 GG		34 (18%)	-
	rs5882 AG/rs708272 AA		10 (5.3%)	-
	rs5882 AG/rs708272 AG		56 (29.6%)	-
	rs5882 AG/rs708272 GG		28 (14.8%)	-
	rs5882 GG/rs708272 AA		7 (3.7%)	-
	rs5882 GG/rs708272 AG		11 (5.8%)	-
	rs5882 GG/rs708272 GG		6 (3.2%)	-

APOE = apolipoprotein E gene; ACE = angiotensin-converting enzyme gene; HMGCR = 3-hydroxy-3-methylglutaryl-CoA reductase gene; LDLR = low-density lipoprotein cholesterol receptor gene; NR1H2 = nuclear receptor 1 type H2 gene (liver X receptor β gene); CETP = cholesteryl ester transfer protein gene.

* Hardy-Weinberg equilibrium (chi-square test).

**Figure 1.**
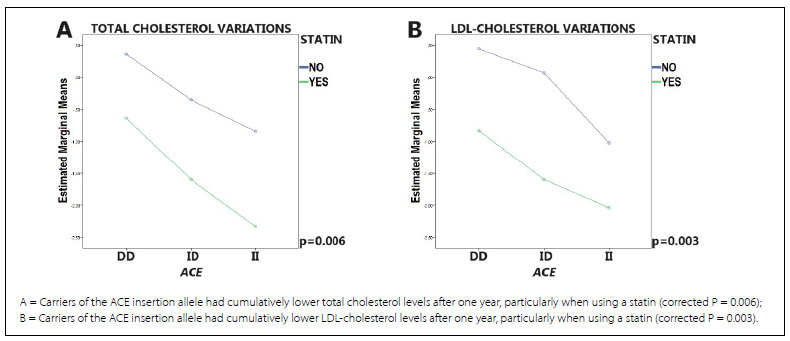
Graphical representations of significant pharmacogenetic findings concerning logarithmically transformed lipid profile variations at one year according to the use or not of a statin and specific genetic variants

Table 3 shows the lipid profile variations at one year according to the indiscriminate use of a statin and specific genetic variants. Overall, statin therapy led to lower total cholesterol and LDL-cholesterol levels after one year along with elevated HDL-cholesterol levels, an effect that did not survive correction for false discovery rates. No significant effects were observed for triglycerides. Carriers of the insertion allele of ACE had cumulatively lower total cholesterol and LDL-cholesterol levels regardless of statin therapy compared to non-carriers (Figure 1). No other genetic variants had any significant effects on lipid profile variations.

**Table 3.** Compared lipid profile variations at one year according to the use or not of a statin and specific genetic variants*

Genetic variants		Total cholesterol variations (mg/dl, mean ± SD)			LDL-cholesterol variations (mg/dl, mean ± SD)		
		Statin n = 140	no statin n = 49	Total n = 189	statin n = 140	no statin n = 49	Total n = 189
APOE haplotypes	APOE-ε4/ε4 n = 21	−40.47 ± 43.5^a1^	−4.4 ± 7.8^a1^	−31.89 ± 41	−36.36 ± 50.6^b1^	−4.96 ± 9.1^b1^	−28.89 ± 46.1
	APOE-ε4+/ε4-, n = 80	−19.68 ± 37.5^a1^	1.83 ± 28^a1^	−13.5 ± 36.2	−20.36 ± 30.2^b1^	2.83 ± 17^b1^	−13.69 ± 29
	APOE-ε4-/ε4-, n = 88	−20.58 ± 39.6^a1^	−1.38 ± 12.2^a1^	−16 ± 35.9	−20.32 ± 35.9^b1^	−1.99 ± 11.9^b1^	−15.94 ± 32.7
ACE Alu repeat insertion/deletion genotypes	I/I, n = 50	−31.2 ± 41.6^a2^	−5.4 ± 10.5^a2^	−23.46 ± 37.1^a2^	−26.9 ± 36^b2^	−5.31 ± 9.4^b2^	−20.42 ± 32^b2^
	I/D, n = 88	−21.58 ± 34.7^a2^	1.60 ± 26.8^a2^	−14.99 ± 34.2^a2^	−21.84 ± 32.9^b2^	1.68 ± 17.2^b2^	−15.16 ± 31.1^b2^
	D/D, n = 51	−16.59 ± 43.8^a2^	3.56 ± 13^a2^	−13.04 ± 40.8^a2^	−18.72 ± 40^b2^	4 ± 10.8^b2^	−14.71 ± 37.5^b2^
LDLR8: rs11669576 genotypes	AA, n = 1	0 ± 0^a3^	-	0 ± 0	0 ± 0^b3^	-	0 ± 0
	AG, n = 28	−29.83 ± 50.2^a3^	−2.6 ± 5.9^a3^	−20.11 ± 42.1	−31.27 ± 44.6^b3^	−3.88 ± 7.5^b3^	−21.49 ± 38.1
	GG, n = 160	−21.58 ± 37.9^a3^	0.44 ± 23.1^a3^	−16.22 ± 36.1	−20.99 ± 34.5^b3^	0.95 ± 15.6^b3^	−15.65 ± 32.3
LDLR10: rs5930 genotypes	AA, n = 23	−21.37 ± 32.4^c3^	−5 ± 16.7^c3^	−17.81 ± 30.2	−21.74 ± 29.3^d3^	−2 ± 13.4^d3^	−17.45 ± 27.7
	AG, n = 83	−23.45 ± 44.3^c3^	−3.21 ± 9.6^c3^	−18.82 ± 40	−25.08 ± 39.2^d3^	−1.09 ± 13^d3^	−19.59 ± 36.3
	GG, n = 83	−21.78 ± 36.2^c3^	3.08 ± 26.9^c3^	−14.29 ± 35.5	−19.09 ± 34^d3^	1.17 ± 15.9^d3^	−12.99 ± 31.1
NR1H2 (intron 2): rs2695121 genotypes	CC, n = 72	−29.19 ± 39.1^a4^	−4.50 ± 8.2^a4^	−25.08 ± 36.9	−26.19 ± 37.1^b4^	0.43 ± 11.5^b4^	−21.75 ± 35.6
	CT, n = 89	−18.37 ± 38.7^a4^	−2.5 ± 12.5^a4^	−13.73 ± 33.9	−19.96 ± 34.6^b4^	−2.63 ± 11.7^b4^	−14.89 ± 30.7
	TT, n = 28	−14.12 ± 42.1^a4^	10 ± 38.2^a4^	−4.64 ± 41.7	−16.18 ± 36.2^b4^	5.60 ± 21.2^b4^	−7.62 ± 32.6
CETP: rs5882 genotypes	AA, n = 71	−12.61 ± 38.7^a5^	1.24 ± 10.9^a5^	−9.29 ± 34.6	−15.22 ± 33.1^b5^	2.64 ± 8.6^b5^	−10.95 ± 30.1
	AG, n = 94	−27.75 ± 39.4^a5^	−0.46 ± 27.2^a5^	−20.2 ± 38.3	−25.36 ± 37.5^b5^	−0.83 ± 18^b5^	−18.58 ± 34.9
	GG, n = 24	−32.25 ± 37.6^a5^	−3 ± 7.3^a5^	−24.94 ± 35	−30.95 ± 35.7^b5^	−4.13 ± 8.4^b5^	−24.24 ± 33.2
CETP: rs708272 genotypes	AA, n = 20	−30.53 ± 36.6^c5^	−6 ± 10.4^c5^	−26.85 ± 34.9	−26.22 ± 33.8^d5^	−7 ± 12.1^d5^	−23.34 ± 32.1
	AG, = 101	−22.46 ± 40.8^c5^	−3.06 ± 8.2^c5^	−15.93 ± 34.7	−23.84 ± 37.5^d5^	−1.51 ± 10.4^d5^	−16.32 ± 32.8
	GG, n = 68	−20.08 ± 39.1^c5^	9.42 ± 39.1^c5^	−14.88 ± 40.4	−18.94 ± 34.7^d5^	5.88 ± 22.1^d5^	−14.56 ± 34
Final variations at one year, n = 189		−22.49 ± 39.4^a^	−0.18 ± 20.8^a^	−16.71 ± 36.8	−22.17 ± 35.8^b^	−0.03 ± 14.4^b^	−16.43 ± 33.1
**Genetic variants**		HDL-cholesterol variations (mg/dl, mean ± SD)			Triglyceride variations (mg/dl, mean ± SD)		
		**statin n = 140**	**no statin n = 49**	**Total n = 189**	**statin n = 140**	**no statin n = 49**	**Total n = 189**
APOE haplotypes	APOE-ε4/ε4 n = 21	−3.59 ± 11.4	−1.6 ± 4.8	−3.11 ± 10.2	−1.9 ± 94.9	10.8 ± 21.5	1.12 ± 83
	APOE-ε4+/ε4-, n = 80	1.12 ± 10.2	−3.78 ± 9.3	−0.29 ± 10.2	−2.3 ± 58.5	14 ± 68.6	2.38 ± 61.6
	APOE-ε4-/ε4-, n = 88	1.54 ± 10	0.57 ± 4.2	1.31 ± 9	−9.22 ± 48	0.14 ± 8	-6.99 ± 42.2
ACE Alu repeat insertion/deletion genotypes	I/I, n = 50	0.63 ± 10.9	−0.8 ± 4.6	0.2 ± 9.4	−24.77 ± 67.1	3.67 ± 20.3	-16.24 ± 58.5
	I/D, n = 88	−0.55 ± 9.4	−2.88 ± 9.5	−1.21 ± 9.4	4.19 ± 49.6	13.72 ± 64.7	6.9 ± 54.1
	D/D, n = 51	2.91 ± 10.9	0.11 ± 2.3	2.41 ± 10.1	−4.21 ± 61.8	−2.11 ± 9.8	-3.84 ± 56.1
LDLR8: rs11669576 genotypes	AA, n = 1	0 ± 0	-	0 ± 0	0 ± 0	−	0 ± 0
	AG, n = 28	−1.5 ± 12.5	0.6 ± 1.7	−0.75 ± 10	14.39 ± 82.8	3.8 ± 12.6	10.61 ± 66.3
	GG, n = 160	1.13 ± 10	−2.28 ± 8.1	0.29 ± 9.7	−8.58 ± 54.5	8.74 ± 53.1	−4.36 ± 54.5
LDLR10: rs5930 genotypes	AA, n = 23	1.31 ± 8.9	−3.4 ± 5.6	0.29 ± 8.5	−4.3 ± 59.9	1.6 ± 6.7	−3.02 ± 52.8
	AG, n = 83	1.27 ± 11.2	−1.68 ± 10.6	0.59 ± 11.1	1.8 ± 61.3	−2.05 ± 9.5	0.92 ± 53.9
	GG, n = 83	0.08 ± 9.8	−1.36 ± 4	−0.35 ± 8.5	−14.09 ± 55.6	16.4 ± 65.6	−4.91 ± 60
NR1H2 (intron 2): rs2695121 genotypes	CC, n = 72	−1.26 ± 9.4^c4^	−6 ± 12.6^c4^	−2.05 ± 10.1	−8.84 ± 60.3	5.33 ± 19.3	−6.48 ± 55.7
	CT, n = 89	1.5 ± 10.7^c4^	−0.04 ± 4.2^c4^	1.05 ± 9.3	0.43 ± 55.9	0.85 ± 9.8	0.55 ± 47.2
	TT, n = 28	5.29 ± 10.7^c4^	−0.91 ± 2.8^c4^	2.86 ± 8.9	−16.24 ± 65.1	26.64 ± 98.6	0.61 ± 81.1
CETP: rs5882 genotypes	AA, n = 71	2.29 ± 10.9	−2.18 ± 6.6	1.23 ± 10.2	1.46 ± 59.8	4.06 ± 15.9	2.08 ± 52.6
	AG, n = 94	0.19 ± 9.6	−2.04 ± 8.5	−0.43 ± 9.3	−12.96 ± 53.9	11.96 ± 64.4	−6.07 ± 57.8
	GG, n = 24	−1.52 ± 11	1.17 ± 2	−0.85 ± 9.6	1.26 ± 72.6	−0.17 ± 3.5	0.9 ± 62.6
CETP: rs708272 genotypes	AA, n = 20	2 ± 10.5	0.67 ± 1.1	1.80 ± 9.7	−32.41 ± 64.7	1.67 ± 2.9	−27.3 ± 60.7
	AG, n = 101	0.64 ± 9.5	−1.44 ± 8.1	−0.06 ± 9.1	3.6 ± 55.9	−0.62 ± 10.4	2.18 ± 45.9
	GG, n = 68	0.58 ± 11.3	−3 ± 5.9	−0.05 ± 10.6	−8.39 ± 58.6	32.92 ± 93.1	−1.1 ± 67
Final variations at one year, n = 189		0.78 ± 10.3^c^	−1.69 ± 7.3^c^	0.14 ± 9.7	−5.57 ± 58.8	7.73 ± 47.6	−2.12 ± 56.3

* General linear model adjusted for sex, baseline age, and body mass index variations at one year. SD = standard deviation; APOE = apolipoprotein E gene: ^a1^ statin versus no statin, uncorrected P = 0.032; ^b1^ statin versus no statin, uncorrected P = 0.030; ACE = angiotensin-converting enzyme gene: ^a2^ genotype effect, statin versus no statin, significant P = 0.006 (corrected for false discovery rates); ^b2^ genotype effect, statin versus no statin, significant P = 0.003 (corrected for false discovery rates); LDLR = low-density lipoprotein cholesterol receptor gene: ^a3^ statin versus no statin, uncorrected P = 0.024; ^b3^ statin versus no statin, uncorrected P = 0.016; ^c3^ statin versus no statin, uncorrected P = 0.032; ^d3^ statin versus no statin, significant P = 0.011 (corrected for false discovery rates); NR1H2 = nuclear receptor 1 type H2 gene (liver X receptor β gene): ^a4^ statin versus no statin, uncorrected P = 0.039; ^b4^ statin versus no statin, significant P = 0.006 (corrected for false discovery rates); ^c4^ statin versus no statin, uncorrected P = 0.036; CETP = cholesteryl ester transfer protein gene: ^a5^ statin versus no statin, uncorrected P = 0.014; ^b5^ statin versus no statin, significant P = 0.005 (corrected for false discovery rates); ^c5^ statin versus no statin, uncorrected P = 0.035; ^d5^ statin versus no statin, uncorrected P = 0.031; Final variations at one year: ^a^ statin versus no statin, significant P = 0.006 (corrected for false discovery rates); ^b^ statin versus no statin, significant P = 0.003 (corrected for false discovery rates); ^c^ statin versus no statin, uncorrected P = 0.036.

Table 4 shows the lipid profile variations at one year according to the use of simvastatin, atorvastatin or no statin and specific genetic variants. Overall, simvastatin therapy resulted in lower total cholesterol and LDL-cholesterol levels, and higher HDL-cholesterol levels after one year, while atorvastatin therapy resulted in lower total cholesterol, LDL-cholesterol and HDL-cholesterol levels after one year. Additionally, atorvastatin therapy resulted in lower triglyceride level variations than simvastatin therapy after one year.

**Table 4.** Compared lipid profile variations at one year according to the use of Simvastatin or Atorvastatin or no statin and specific genetic variants*

Genetic variants		Total cholesterol variations (mg/dl, mean ± SD)			LDL-cholesterol variations (mg/dl, mean ± SD)		
		Simvastatin, n = 124	Atorvastatin, n = 14	no statin, n = 49	Simvastatin, n = 124	Atorvastatin, n = 14	no statin, n = 49
APOE haplotypes	APOE-ε4/ε4, n = 21	−40.47 ± 43.5^a1^	-	−4.40 ± 7.8^a1^	−36.36 ± 50.6^b1^	-	−4.96 ± 9.1^b1^
	APOE-ε4+/ε4-, n = 78	−23.19 ± 37.3^a1^	0.5 ± 7.3	1.83 ± 28^a1^	−22.94 ± 30.8^b1^	−3.25 ± 4.1	2.83 ± 17^b1^
	APOE-ε4-/ε4-, n = 88	−17.72 ± 39.4^a1^	−36.9 ± 38.6	−1.38 ± 12.2^a1^	−19.46 ± 35.4^b1^	−25.2 ± 40.4	−1.99 ± 11.9^b1^
ACE Alu repeat insertion/deletion genotypes	I/I, n = 50	−32.06 ± 42.8^a2^	−22 ± 29.9	−5.4 ± 10.5^a2^	−28.99 ± 36.4^b2^	−4.67 ± 26.6	−5.31 ± 9.4^b2^
	I/D, n = 88	−20.5 ± 34.6^a2^	−29 ± 36.5	1.6 ± 26.8^a2^	−21.93 ± 32.7^b2^	−21.25 ± 36.8	1.68 ± 17.2^b2^
	D/D, n = 49	−18.57 ± 42.9^a2^	−23 ± 56.3	3.56 ± 13^a2^	−19.66 ± 40.5^b2^	−27 ± 46.8	4 ± 10.8^b2^
LDLR8: rs11669576 genotypes	AA, n = 1	0 ± 0	-	-	0 ± 0	-	-
	AG, n = 28	−26.65 ± 49.8^a3^	−84 ± 0^b3^	−2.6 ± 5.9^a3,b3^	−30.05 ± 45.7^c3^	−52 ± 0^d3^	−3.88 ± 7.5^c3,d3^
	GG, n = 158	−22.52 ± 37.9^a3^	−21.77 ± 34.2^b3^	0.44 ± 23.1^a3,b3^	−22.17 ± 34.5^c3^	−16.38 ± 35.3^d3^	0.95 ± 15.6^c3,d3^
LDLR10: rs5930 genotypes	AA, n = 23	−23.54 ± 33.9^f3^	−13.75 ± 29.4^g3^	−5 ± 16.7^f3,g3^	−26.52 ± 30.2^h3^	−5 ± 20.5	−2 ± 13.4^h3^
	AG, n = 82	−24.63 ± 43.9^f3^	−26.75 ± 40.9^g3^	−3.21 ± 9.6^f3,g3^	−26.29 ± 39.1^h3^	−19 ± 42.9	−1.09 ± 13.0^h3^
	GG, n = 82	−20.75 ± 35.9^f3^	−34.17 ± 42.3^g3^	3.08 ± 26.9^f3,g3^	−18.4 ± 33.7^h3^	−28.17 ± 40.1	1.17 ± 15.9^h3^
NR1H2 (intron 2): rs2695121 genotypes	CC, n = 72	−29.81 ± 38.9^a4^	−26.1 ± 41.7^b4^	−4.5 ± 8.2^a4,b4^	−27.68 ± 36.4^c4^	−18.7 ± 41.5	0.43 ± 11.5^c4^
	CT, n = 87	−19.83 ± 38.5^a4^	−22 ± 29.4^b4^	−2.5 ± 12.5^a4,b4^	−21.22 ± 35.4^c4^	−16.33 ± 15.3	−2.63 ± 11.7^c4^
	TT, n = 28	−12.5 ± 42.9^a4^	−40 ± 0.0^b4^	10 ± 38.2^a4,b4^	−15.38 ± 37.3^c4^	−29 ± 0	5.60 ± 21.2^c4^
CETP: rs5882 genotypes	AA, n = 70	−13.98 ± 39.0^a5^	−13.75 ± 17.9^b5^	1.24 ± 10.9^a5,b5^	−16.88 ± 33.8^c5^	−4.25 ± 19.7^d5^	2.64 ± 8.6^c5,d5^
	AG, n = 93	−28.2 ± 39.6^a5^	−27.87 ± 41.7^b5^	−0.46 ± 27.2^a5,b5^	−26.03 ± 37.3^c5^	−23.62 ± 42.6	−0.83 ± 18.0^c5^
	GG, n = 24	−30.72 ± 37.1^a5^	−44.5 ± 55.9^b5^	−3 ± 7.3^a5,b5^	−31.13 ± 37.1^c5^	−29.5 ± 31.8^d5^	−4.13 ± 8.4^c5,d5^
CETP: rs708272 genotypes	AA, n = 20	−31.69 ± 36.8^f5^	−26.75 ± 40.9^g5^	−6 ± 10.4^f5,g5^	−28.14 ± 32.7^h5^	−20 ± 41.8	−7 ± 12.1^h5^
	AG, n = 100	−23.64 ± 39.9^f5^	−24.4 ± 43.5^g5^	−3.06 ± 8.2^f5,g5^	−24.47 ± 37.4^h5^	−25.4 ± 41.7	−1.51 ± 10.4^h5^
	GG, n = 67	−19.74 ± 39.9^f5^	−27.6 ± 35.3^g5^	9.42 ± 39.1^f5,g5^	−20.05 ± 35.6^h5^	−11.6 ± 29.3	5.88 ± 22.1^h5^
Final variations at one year, n = 187		−22.91 ± 39.5^a^	−26.21 ± 36.8^b^	−0.18 ± 20.8^a,b^	−23.07 ± 36.0^c^	−18.93 ± 35.2^d^	−0.03 ± 14.4^c,d^
**Genetic variants**		**HDL-cholesterol variations (mg/dl, mean ± SD)**			**Triglyceride variations (mg/dl, mean ± SD)**		
		**Simvastatin, n = 124**	**Atorvastatin, n = 14**	**no statin, n = 49**	**Simvastatin, n = 124**	**Atorvastatin, n = 14**	**no statin, n = 49**
APOE haplotypes	APOE-ε4/ε4, n = 21	−3.59 ± 11.4	-	−1.6 ± 4.8	−1.9 ± 94.9	-	10.8 ± 21.5
	APOE-ε4+/ε4-, n = 78	0.17 ± 9.2	4 ± 4.2^c1^	−3.78 ± 9.3^c1^	−2.18 ± 61.7	−2.5 ± 23.8	14 ± 68.6
	APOE-ε4-/ε4-, n = 88	2.54 ± 9.9	−4.2 ± 9.3^c1^	0.57 ± 4.2^c1^	−4.07 ± 44.3	−38.6 ± 59.9	0.14 ± 8
ACE Alu repeat insertion/deletion genotypes	I/I, n = 50	0.91 ± 11.4	−2.33 ± 1.2	−0.8 ± 4.6	−19.88 ± 66.3^c2^	−77 ± 63.4^c2,d2^	3.67 ± 20.3^d2^
	I/D, n = 88	−0.3 ± 9.3	−2.25 ± 10.5	−2.88 ± 9.5	8.88 ± 48.6^c2^	−28 ± 46.7^c2,d2^	13.72 ± 64.7^d2^
	D/D, n = 49	2.27 ± 9.6	−0.33 ± 11	0.11 ± 2.3	−6.11 ± 65.4^c2^	19.67 ± 13.6^c2,d2^	−2.11 ± 9.8^d2^
LDLR8: rs11669576 genotypes	AA, n = 1	0 ± 0	-	-	0 ± 0	-	-
	AG, n = 28	−0.53 ± 12.1	−18 ± 0	0.6 ± 1.7	19.35 ± 82.5	−70 ± 0^e3^	3.8 ± 12.6^e3^
	GG, n = 158	0.99 ± 9.7	−0.62 ± 7.9	−2.28 ± 8.1	−6.63 ± 54.9	−25.08 ± 54.6^e3^	8.74 ± 53.1^e3^
LDLR10: rs5930 genotypes	AA, n = 23	1.47 ± 10	0.75 ± 4.3	−3.4 ± 5.6	8.54 ± 52.1	−49.25 ± 71.7	1.6 ± 6.7
	AG, n = 82	0.81 ± 10.4	−1.5 ± 8.9	−1.68 ± 10.6	4.37 ± 61.2	−33.25 ± 67.3	−2.05 ± 9.5
	GG, n = 82	0.55 ± 9.6	−3.83 ± 11.7	−1.36 ± 4.0	−14.73 ± 58.4	−11 ± 31.7	16.40 ± 65.6
NR1H2 (intron 2): rs2695121 genotypes	CC, n = 72	−0.95 ± 9.4^d4^	−2.8 ± 9.7	−6 ± 12.6^d4^	−5.73 ± 60.5	−24.4 ± 59.9^f4^	5.33 ± 19.3^f4^
	CT, n = 87	0.79 ± 9.9^d4^	3.33 ± 5.7^e4^	−0.04 ± 4.2^d4,e4^	2.98 ± 56.7	−45.33 ± 45.9^f4^	0.85 ± 9.8^f4^
	TT, n = 28	6.12 ± 10.5^d4^	−8 ± 0^e4^	−0.91 ± 2.8^d4,e4^	−16.25 ± 67.3	−16 ± 0^f4^	26.64 ± 98.6^f4^
CETP: rs5882 genotypes	AA, n = 70	1.88 ± 9.9	−1.75 ± 5.4	−2.18 ± 6.6	5.06 ± 59.7	−39.75 ± 58.9^e5^	4.06 ± 15.9^e5^
	AG, n = 93	0.37 ± 9.8	−1.12 ± 9.1	−2.04 ± 8.5	−12.61 ± 54.3	−17.12 ± 58.5^e5^	11.96 ± 64.4^e5^
	GG, n = 24	−1.09 ± 10.6	−5 ± 18.4	1.17 ± 2.0	7.66 ± 74.4	−50 ± 28.3^e5^	−0.17 ± 3.5^e5^
CETP: rs708272 genotypes	AA, n = 20	2.85 ± 11.2	−0.75 ± 8.8^i5^	0.67 ± 1.1^i5^	−32.62 ± 67.4	−31.75 ± 64.5^j5^	1.67 ± 2.9^j5^
	AG, n = 100	−0.21 ± 8.5	3.4 ± 5.2^i5^	−1.44 ± 8.1^i5^	5.24 ± 57.1	−13.6 ± 42.3^j5^	−0.62 ± 10.4^j5^
	GG, n = 67	1.45 ± 11.3	−8 ± 9.5^i5^	−3 ± 5.9^i5^	−5.38 ± 58.8	−40.2 ± 59.2^j5^	32.92 ± 93.1^j5^
Final variations at one year, n = 187		0.78 ± 9.9^e^	−1.86 ± 8.9^f^	−1.69 ± 7.3^e,f^	−3.01 ± 59.5^g^	−28.29 ± 53.8^g,h^	7.73 ± 47.6^h^

*General linear model adjusted for sex, baseline age, and body mass index variations at one year. SD = standard deviation; APOE = apolipoprotein E gene: ^a1^Simvastatin versus no statin, significant P = 0.004 (corrected for false discovery rates); ^b1^Simvastatin versus no statin, significant P = 0.019 (corrected for false discovery rates); ^c1^APOE-ε4+/ε4- versus APOE-ε4-/ε4-, Atorvastatin versus no statin, significant P = 0.011 (corrected for false discovery rates); ACE = angiotensin-converting enzyme gene: ^a2^ genotype effect, Simvastatin versus no statin, significant P = 0.008 (corrected for false discovery rates); ^b2^genotype effect, Simvastatin versus no statin, significant P = 0.003 (corrected for false discovery rates); ^c2^genotype effect, Simvastatin versus Atorvastatin, significant P = 0.029 (corrected for false discovery rates); ^d2^genotype effect, Atorvastatin versus no statin, significant P = 0.001 (corrected for false discovery rates); LDLR = low-density lipoprotein cholesterol receptor gene: ^a3^AG versus GG, Simvastatin versus no statin, significant P = 0.032 (corrected for false discovery rates); ^b3^AG versus GG, Atorvastatin versus no statin, significant P = 0.011 (corrected for false discovery rates); ^c3^AG versus GG, Simvastatin versus no statin, significant P = 0.016 (corrected for false discovery rates); ^d3^AG versus GG, Atorvastatin versus no statin, uncorrected P = 0.036; ^e3^AG versus GG, Atorvastatin versus no statin, significant P = 0.019 (corrected for false discovery rates); ^f3^Simvastatin versus no statin, significant P = 0.024 (corrected for false discovery rates); ^g3^Atorvastatin versus no statin, significant P = 0.027 (corrected for false discovery rates); ^h3^Simvastatin versus no statin, significant P = 0.006 (corrected for false discovery rates); NR1H2 = nuclear receptor 1 type H2 gene (liver X receptor β gene): ^a4^ genotype effect, Simvastatin versus no statin, uncorrected P = 0.033; ^b4^ Atorvastatin versus no statin, significant P = 0.019 (corrected for false discovery rates); ^c4^ Simvastatin versus no statin, significant P = 0.005 (corrected for false discovery rates); ^d4^Simvastatin versus no statin, significant P = 0.026 (corrected for false discovery rates); ^e4^CT versus TT, Atorvastatin versus no statin, uncorrected P = 0.040; ^f4^Atorvastatin versus no statin, uncorrected P = 0.041; CETP = cholesteryl ester transfer protein gene: ^a5^Simvastatin versus no statin, significant P = 0.015 (corrected for false discovery rates); ^b5^Atorvastatin versus no statin, significant P = 0.013 (corrected for false discovery rates); ^c5^Simvastatin versus no statin, significant P = 0.004 (corrected for false discovery rates); ^d5^AA versus GG, Atorvastatin versus no statin, significant P = 0.032 (corrected for false discovery rates); ^e5^genotype effect, Atorvastatin versus no statin, significant P = 0.014 (corrected for false discovery rates); ^f5^Simvastatin versus no statin, uncorrected P = 0.034; ^g5^Atorvastatin versus no statin, significant P = 0.022 (corrected for false discovery rates); ^h5^Simvastatin versus no statin, significant P = 0.021 (corrected for false discovery rates); ^i5^genotype effect, Atorvastatin versus no statin, significant P = 0.020 (corrected for false discovery rates); ^j5^Atorvastatin versus no statin, uncorrected P = 0.035; Final variations at one year: ^a^Simvastatin versus no statin, significant P = 0.008 (corrected for false discovery rates); ^b^Atorvastatin versus no statin, significant P = 0.013 (corrected for false discovery rates); ^c^Simvastatin versus no statin, significant P = 0.003 (corrected for false discovery rates); ^d^Atorvastatin versus no statin, significant P = 0.032 (corrected for false discovery rates); ^e^Simvastatin versus no statin, significant P = 0.026 (corrected for false discovery rates); ^f^Atorvastatin versus no statin, significant P = 0.011 (corrected for false discovery rates); ^g^Simvastatin versus Atorvastatin, significant P = 0.029 (corrected for false discovery rates); ^h^ Atorvastatin versus no statin, uncorrected P = 0.035.

Regarding genetic variants, the following findings remained significant after corrections for false discovery rates (Figure 2): APOE-ε4 carriers had a better response to atorvastatin, in terms of elevated HDL-cholesterol levels, than APOE-ε4 non-carriers. Carriers of the insertion allele of ACE had cumulatively lower total cholesterol and LDL-cholesterol levels when using simvastatin than when using atorvastatin. Conversely, they had cumulatively lower triglyceride levels when using atorvastatin than when using simvastatin, although carriers of the D/D genotype exhibited elevated triglyceride levels with atorvastatin. Carriers of the insertion allele of ACE had cumulatively lower triglycerides when using atorvastatin in comparison to those who used simvastatin, although carriers of the D/D genotype exhibited lower triglycerides only when using simvastatin. Carriers of the G allele of rs11669576 (LDLR8) had lower total cholesterol and LDL cholesterol levels with simvastatin, but lower total cholesterol and triglyceride levels with atorvastatin. Carriers of the GG genotype of rs5882 (CETP) had lower LDL-cholesterol levels with atorvastatin than carriers of the AA genotype using a statin or not. Carriers of the AA and GG genotypes of rs5882 (CETP) had lower triglyceride levels with atorvastatin than with simvastatin, although the effects were stronger for GG carriers. Carriers of the A allele of rs708272 (CETP) had higher increases in HDL cholesterol levels with atorvastatin, whereas carriers of the GG genotype had lower HDL cholesterol levels with atorvastatin.

**Figure 2.**
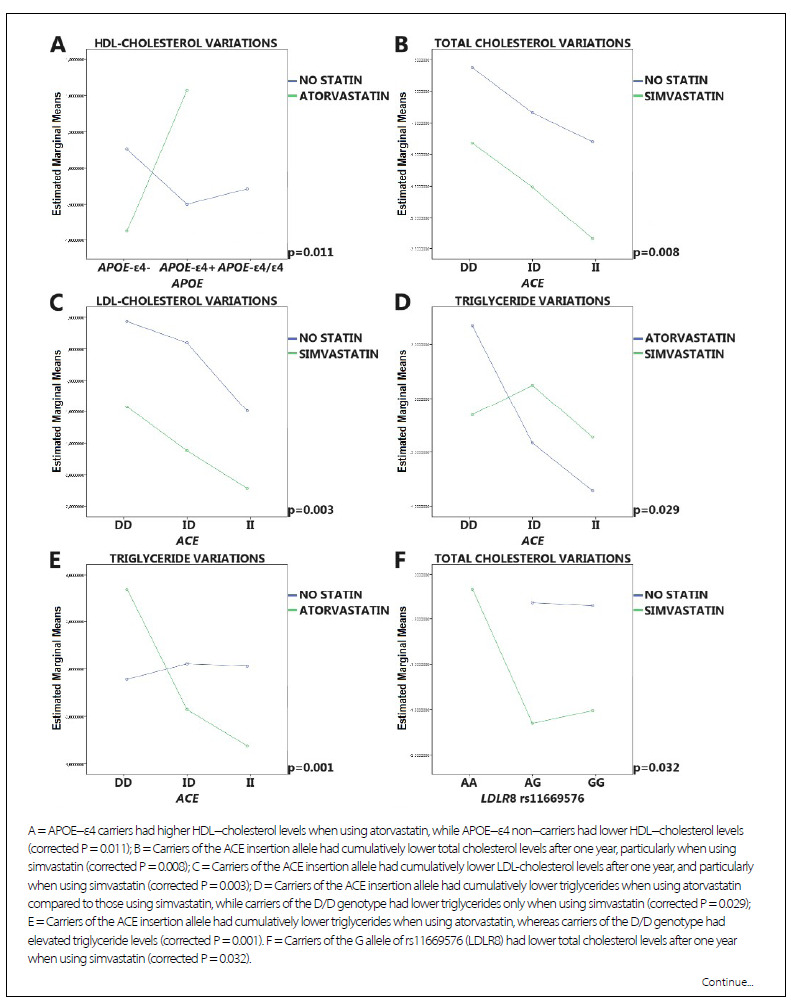
Graphical representations of significant pharmacogenetic findings concerning logarithmically transformed lipid profile variations at one year according to the use or not of simvastatin or atorvastatin and specific genetic variants

**Figure 2.**
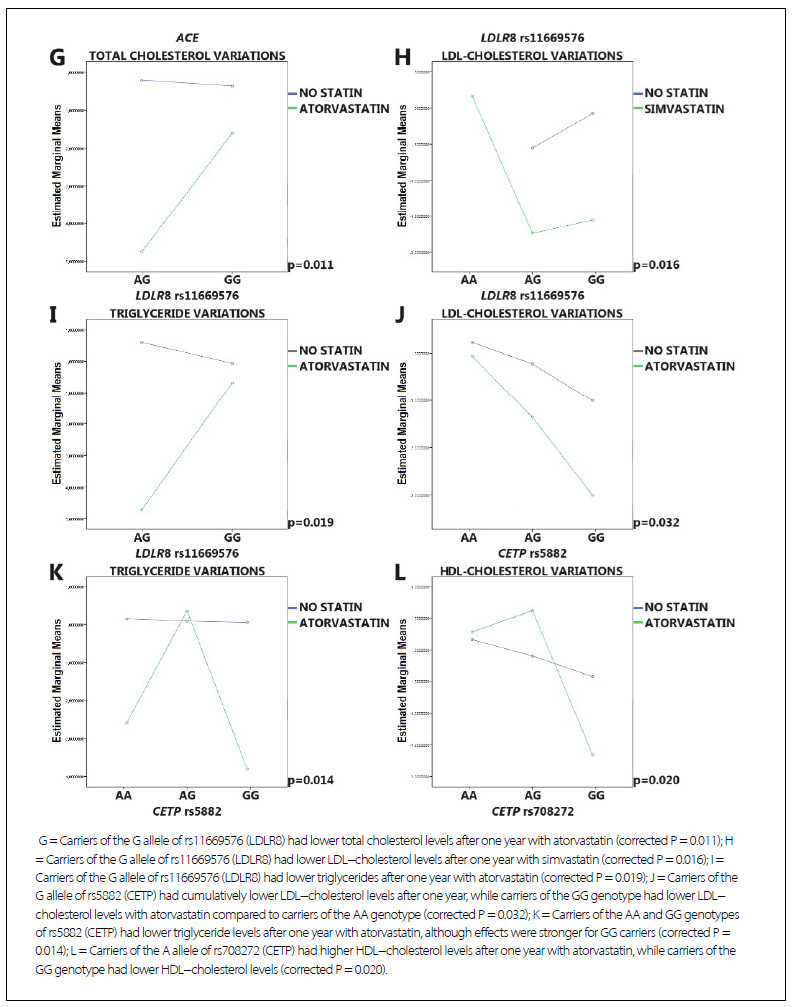
Continuation.

Table 5 shows the lipid profile variations at one year according to the indiscriminate use of a statin or not and specific CETP haplotypes. Statin therapy resulted in lower total cholesterol and LDL-cholesterol levels after one year. Notably, carriers of CETP haplotypes with the A allele of rs5882 and the A allele of rs708272 benefitted the most from statins in terms of lower total cholesterol and elevated HDL-cholesterol levels.

**Table 5.** Compared lipid profile variations at one year according to the use or not of a statin and specific CETP haplotypes*

CETP haplotypes		Total cholesterol variations (mg/dl, mean ± SD)			LDL-cholesterol variations (mg/dl, mean ± SD)		
		statin n = 140	no statin, n = 49	Total, n = 189	statin, n = 140	no statin, n = 49	Total, n = 189
rs5882 AA/rs708272 AA	Yes, n = 3	−58 ± 0	0 ± 0	−19.33 ± 33.5	−26 ± 0	0 ± 0	−8.67 ± 15
	No, n = 186	−22.23 ± 39.5	−0.19 ± 21.2	−16.66 ± 36.9	−22.14 ± 35.9	−0.03 ± 14.7	−16.56 ± 33.3
rs5882 AA/rs708272 AG	Yes, n = 34	−11.92 ± 36.1	−0.33 ± 6.6	−8.85 ± 31.3	−17.72 ± 30.9^a^	1.62 ± 4.5^a^	−12.6 ± 27.8
	No, n = 155	−24.79 ± 39.9	−0.15 ± 22.9	−18.43 ± 37.8	−23.14 ± 36.9^a^	−0.41 ± 15.8^a^	−17.27 ± 34.2
rs5882 AA/rs708272 GG	Yes, n = 34	−11.61 ± 41.3	4 ± 17.3	−8.85 ± 38.4	−12.61 ± 35.8^b^	5.03 ± 13.9^b^	−9.49 ± 33.6
	No, n = 155	−25.21 ± 38.7	−0.77 ± 21.4	−18.43 ± 36.4	−24.56 ± 35.6^b^	−0.74 ± 14.4^b^	−17.95 ± 32.9
rs5882 AG/rs708272 AA	Yes, n = 10	−36.8 ± 41^c^	-	−36.8 ± 41	−32.08 ± 41.8	-	−32.08 ± 41.8
	No, n = 179	−21.39 ± 39.3^c^	−0.18 ± 20.8^c^	−15.58 ± 36.4	−21.41 ± 35.4^d^	−0.03 ± 14.4^d^	−15.56 ± 32.5
rs5882 AG/rs708272 AG	Yes, n = 56	−25.49 ± 42.7^e^	−5.05 ± 9.5^e^	−18.19 ± 35.9	−22.59 ± 38.1^f^	−3.1 ± 13.2^f^	−15.63 ± 32.7
	No, n = 133	−21.45 ± 38.4^e^	3.17 ± 25.5^e^	−16.08 ± 37.4	−22.02 ± 35.2^f^	2.08 ± 14.9^f^	−16.77 ± 33.4
rs5882 AG/rs708272 GG	Yes, n = 28	−27.32 ± 33.9^g^	14.83 ± 54.7^g^	−18.29 ± 41.9	−26.84 ± 35.7^h^	6.73 ± 29.6^h^	−19.64 ± 36.7
	No, n = 161	−21.59 ± 40.5^g^	−2.28 ± 10.1^g^	−16.43 ± 36	−21.30 ± 35.9^h^	−0.98 ± 11.1^h^	−15.87 ± 32.5
rs5882 GG/rs708272 AA	Yes, n = 7	−15.5 ± 27.3	−18 ± 0	−15.86 ± 24.9	−16.5 ± 18.5	−21 ± 0	−17.14 ± 16.9
	No, n = 182	−22.8 ± 39.9	0.19 ± 20.8	−16.74 ± 37.3	−22.42 ± 36.4	0.4 ± 14.2	−16.4 ± 33.6
rs5882 GG/rs708272 AG	Yes, n = 11	−48.17 ± 39.5^i^	0 ± 0^i^	−26.27 ± 37.6	−56.83 ± 48.3^j^	−0.76 ± 1.7^j^	−31.35 ± 45
	No, n = 178	−21.34 ± 39.2^i^	−0.2 ± 21.9^i^	−16.12 ± 36.8	−20.62 ± 34.6^j^	0.05 ± 15.2^j^	−15.51 ± 32.2
rs5882 GG/rs708272 GG	Yes, n = 6	−33.09 ± 43.2	-	−33.09 ± 43.2	−19.51 ± 21.2	-	−19.51 ± 21.2
	No, n = 183	−22.01 ± 39.4^k^	−0.18 ± 20.8^k^	−16.17 ± 36.6	−22.29 ± 36.4^l^	−0.03 ± 14.4^l^	−16.33 ± 33.5
CETP haplotypes		HDL-cholesterol variations (mg/dl, mean ± SD)			Triglyceride variations (mg/dl, mean ± SD)		
		statin, n = 140	no statin, n = 49	Total, n = 189	statin, n = 140	no statin, n = 49	Total, n = 189
rs5882 AA/rs708272 AA	Yes, n = 3	13 ± 0	0 ± 0	4.33 ± 7.5	−226 ± 0	0 ± 0	−75.33 ± 130.5
	No, n = 186	0.69 ± 10.3	−1.77 ± 7.5	−0.07 ± 9.7	−3.98 ± 55.9	8.06 ± 48.6	−0.94 ± 54.3
rs5882 AA/rs708272 AG	Yes, n = 34	2.68 ± 10.6	−2.22 ± 7.4	1.38 ± 9.9	15.40 ± 46.6	1.11 ± 7.8	11.62 ± 40.5
	No, n = 155	0.37 ± 10.3	−1.58 ± 7.4	−0.13 ± 9.6	−10.13 ± 60.4	9.23 ± 52.6	−5.13 ± 58.9
rs5882 AA/rs708272 GG	Yes, n = 34	1.57 ± 11.3	−2.83 ± 6.9	0.79 ± 10.7	−2.86 ± 54.4	9.83 ± 25.5	−0.62 ± 50.5
	No, n = 155	0.58 ± 10.1	−1.53 ± 7.4	−0.01 ± 9.5	−6.25 ± 60.1	7.44 ± 50.2	−2.45 ± 57.7
rs5882 AG/rs708272 AA	Yes, n = 10	−0.6 ± 10.5	-	−0.6 ± 10.5	−21.4 ± 51.4	-	−21.4 ± 51.4
	No, n = 179	0.89 ± 10.3	−1.69 ± 7.3	0.18 ± 9.7	−4.35 ± 59.4	7.73 ± 47.7	−1.04 ± 56.5
rs5882 AG/rs708272 AG	Yes, n = 56	−0.66 ± 9.1	−1.70 ± 9.3	−1.03 ± 9.1	−11.18 ± 50.1	−1.25 ± 12.6	−7.63 ± 40.9
	No, n = 133	1.28 ± 10.7	−1.69 ± 5.7	0.63 ± 9.9	−3.63 ± 61.7	13.93 ± 60.7	0.2 ± 61.7
rs5882 AG/rs708272 GG	Yes, n = 28	1.94 ± 10.3	−3.17 ± 5.4	0.85 ± 9.6	−12.03 ± 62.7	56 ± 130.9	2.55 ± 83.9
	No, n = 161	0.57 ± 10.4	−1.49 ± 7.6	0.02 ± 9.7	−4.36 ± 58.3	1 ± 13.3	−2.93 ± 50.4
rs5882 GG/rs708272 AA	Yes, n = 7	4.5 ± 10.5	2 ± 0	4.14 ± 9.7	−18.5 ± 25.9	5 ± 0	−15.14 ± 25.3
	No, n = 182	0.62 ± 10.3	−1.77 ± 7.4	−0.01 ± 9.7	−4.99 ± 59.9	7.79 ± 48.2	−1.62 ± 57.2
rs5882 GG/rs708272 AG	Yes, n = 11	0 ± 6.8	1 ± 2.2	0.45 ± 5.1	43.17 ± 96	−1.2 ± 2.7	23 ± 71.8
	No, n = 178	0.82 ± 10.5	−2 ± 7.6	0.12 ± 9.9	−7.75 ± 56.2	8.75 ± 50.2	−3.67 ± 55.1
rs5882 GG/rs708272 GG	Yes, n = 6	−9.06 ± 11.9^m^	-	−9.06 ± 11.9	−20.9 ± 69.8	-	−20.9 ± 69.8
	No, n = 183	1.22 ± 10.1^m^	−1.69 ± 7.3^m^	0.44 ± 9.5	−4.88 ± 58.5	7.73 ± 47.7	−1.51 ± 55.9

* General linear model adjusted for sex, baseline age, and body mass index variations at one year. SD: standard deviation. CETP: Cholesteryl ester transfer protein ^a^ statin versus no statin, P = 0.019 (corrected for false discovery rates). ^b^ statin versus no statin, uncorrected P = 0.047. ^c^ haplotype effect, statin versus no statin, significant P = 0.004 (corrected for false discovery rates). ^d^ statin versus no statin, P = 0.008 (corrected for false discovery rates). ^e^ statin versus no statin, P = 0.042 (corrected for false discovery rates). ^f^ statin versus no statin, significant P = 0.015 (corrected for false discovery rates). ^g^ statin versus no statin, P = 0.035 (corrected for false discovery rates). ^h^ statin versus no statin, P = 0.031 (corrected for false discovery rates). ^i^ statin versus no statin, P = 0.038 (corrected for false discovery rates). ^j^ statin versus no statin, significant P = 0.027 (corrected for false discovery rates). ^k^ statin versus no statin, P = 0.019 (corrected for false discovery rates). ^l^ statin vs. no statin, P = 0.008 (corrected for false discovery rates). ^m^ Haplotype effect, statin versus no statin, P = 0.046 (corrected for false discovery rates)

Table 6 shows the lipid profile variations at one year according to the use of simvastatin, atorvastatin or no statin and specific CETP haplotypes. Simvastatin therapy resulted in lower total cholesterol and LDL-cholesterol levels for most patients after one year, while atorvastatin therapy resulted in lower triglyceride, total cholesterol and LDL-cholesterol levels for most patients after one year. Carriers of CETP haplotypes with the A allele of rs5882 and the A allele of rs708272 benefitted mostly from atorvastatin, which lowered triglyceride levels. Conversely, the effects of atorvastatin lowering total cholesterol and LDL-cholesterol levels were more pronounced for carriers of rs5882 GG/rs708272 GG. No significant effects were found for changes in HDL-cholesterol levels after one year.

**Table 6.** Compared lipid profile variations at one year according to the use of Simvastatin or Atorvastatin or no statin and specific CETP haplotypes*

CETP haplotypes		Total cholesterol variations (mg/dl, mean ± SD)			LDL−cholesterol variations (mg/dl, mean ± SD)		
		Simvastatin, n = 124	Atorvastatin, n = 14	no statin, n = 49	Simvastatin, n = 124	Atorvastatin, n = 14	no statin, n = 49
rs5882 AA/rs708272 AA	Yes, n = 3	−58 ± 0	-	0 ± 0	−26 ± 0	-	0 ± 0
	No, n = 184	−22.62 ± 39.5	-	−0.19 ± 21.2	−23.05 ± 36.2	-	−0.03 ± 14.7
rs5882 AA/rs708272 AG	Yes, n = 34	−15.52 ± 34.2^a^	0 ± 0	−0.33 ± 6.6^a^	−20.31 ± 30.8^b^	2 ± 0	1.62 ± 4.5^b^
	No, n = 153	−24.59 ± 40.5^a^	−28.23 ± 37.5	−0.15 ± 22.9^a^	−23.70 ± 37.2^b^	−20.54 ± 36.1	−0.41 ± 15.8^b^
rs5882 AA/rs708272 GG	Yes, n = 34	−10.80 ± 43.4	−18.33 ± 18.9^c^	4 ± 17.3^c^	−13.36 ± 37.3^d^	−6.33 ± 23.5	5.03 ± 13.9^d^
	No, n = 153	−25.97 ± 38	−28.36 ± 40.8^c^	−0.77 ± 21.4^c^	−25.52 ± 35.5^d^	−22.36 ± 37.9	−0.74 ± 14.4^d^
rs5882 AG/rs708272 AA	Yes, n = 10	−38 ± 42.3^f^	−34 ± 46.9^g^	-	−35.40 ± 41.7^h^	−24.33 ± 50.1	-
	No, n = 177	−22 ± 39.3^f^	−24.09 ± 36^g^	−0.18 ± 20.8^f,g^	−22.33 ± 35.7^h^	−17.45 ± 33.2	−0.03 ± 14.4^h^
rs5882 AG/rs708272 AG	Yes, n = 56	−24.87 ± 42.8^i^	−30.50 ± 47.7^j^	−5.05 ± 9.5^i,j^	−21.39 ± 37.8^k^	−32.25 ± 44.8	−3.10 ± 13.2^k^
	No, n = 131	−22.22 ± 38.4^i^	−24.50 ± 34.4^j^	3.17 ± 25.5^i,j^	−23.66 ± 35.6^k^	−13.60 ± 31.8	2.08 ± 14.9^k^
rs5882 AG/rs708272 GG	Yes, n = 28	−30.11 ± 34.4^m^	1 ± 0	14.83 ± 54.7^m^	−30.17 ± 35.7^n^	13 ± 0	6.73 ± 29.6^n^
	No, n = 159	−21.52 ± 40.4^m^	−28.31 ± 37.4	−2.28 ± 10.1^m^	−21.71 ± 36.1^n^	−21.38 ± 35.4	−0.98 ± 11.1^n^
rs5882 GG/rs708272 AA	Yes, n = 7	−17.60 ± 29.9	−5 ± 0	−18 ± 0	−18.40 ± 20	−7 ± 0	−21 ± 0
	No, n = 180	−23.13 ± 39.9	−27.85 ± 37.8	0.19 ± 20.8	−23.27 ± 36.6	−19.85 ± 36.5	0.40 ± 14.2
rs5882 GG/rs708272 AG	Yes, n = 10	−48.17 ± 39.5^p^	-	0 ± 0^p^	−56.83 ± 48.3^q^	-	−0.76 ± 1.7^q^
	No, n = 177	−21.62 ± 39.2^p^	-	−0.20 ± 21.9^p^	−21.36 ± 34.7^q^	-	0.05 ± 15.2^q^
rs5882 GG/rs708272 GG	Yes, n = 5	−22.92 ± 39.4^r^	−84 ± 0^s^	-	−13.01 ± 15.7^t^	−52 ± 0^u^	−
	No, n = 182	−22.91 ± 39.6^r^	−21.77 ± 34.2^s^	−0.18 ± 20.8^r,s^	−23.49 ± 36.6^t^	−16.38 ± 35.3^u^	−0.03 ± 14.4^t,u^
***CETP* haplotypes**		HDL−cholesterol variations mg/dl, mean ± SD)			Triglyceride variations (mg/dl, mean ± SD)		
		**Simvastatin, n = 124**	**Atorvastatin, n = 14**	**no statin, n = 49**	**Simvastatin, n = 124**	**Atorvastatin, n = 14**	**no statin, n = 49**
rs5882 AA/rs708272 AA	Yes, n = 3	13 ± 0	-	0 ± 0	−226 ± 0	-	0 ± 0
	No, n = 184	0.68 ± 9.9	-	−1.77 ± 7.5	−1.20 ± 56.2	-	8.06 ± 48.6
rs5882 AA/rs708272 AG	Yes, n = 34	1.26 ± 7.7	−1 ± 0	−2.22 ± 7.4	17.43 ± 48.1	−6 ± 0	1.11 ± 7.8
	No, n = 153	0.67 ± 10.4	−1.92 ± 9.3	−1.58 ± 7.4	−7.67 ± 61	−30 ± 55.6	9.23 ± 52.6
rs5882 AA/rs708272 GG	Yes, n = 34	2 ± 11.8	−2 ± 6.6	−2.83 ± 6.9	2.92 ± 51.3	−51 ± 66.8^e^	9.83 ± 25.5^e^
	No, n = 153	0.47 ± 9.5	−1.82 ± 9.7	−1.53 ± 7.4	−4.51 ± 61.5	−22.09 ± 51.8^e^	7.44 ± 50.2^e^
rs5882 AG/rs708272 AA	Yes, n = 10	0.71 ± 11.7	−3.67 ± 8.1	-	−16.71 ± 42.5	−32.33 ± 79	-
	No, n = 177	0.78 ± 9.9	−1.36 ± 9.4	−1.69 ± 7.3	−2.19 ± 60.4	−27.18 ± 50.1	7.73 ± 47.7
rs5882 AG/rs708272 AG	Yes, n = 56	−1.31 ± 9.3	4.50 ± 5.3	−1.70 ± 9.3	−10.64 ± 50.3	−15.50 ± 55.5^l^	−1.25 ± 12.6^l^
	No, n = 131	1.50 ± 10.1	−4.40 ± 8.9	−1.69 ± 5.7	−0.36 ± 62.4	−33.40 ± 55.3^l^	13.93 ± 60.7^l^
rs5882 AG/rs708272 GG	Yes, n = 28	2.94 ± 9.9	−16 ± 0	−3.17 ± 5.4	−14.34 ± 65.3	22 ± 0^o^	56 ± 130.9^o^
	No, n = 159	0.36 ± 9.9	−0.77 ± 8.2	−1.49 ± 7.6	−0.84 ± 58.4	−32.15 ± 54^o^	1 ± 13.3^o^
rs5882 GG/rs708272 AA	Yes, n = 7	3.80 ± 11.6	8 ± 0	2 ± 0	−16.20 ± 28.3	−30 ± 0	5 ± 0
	No, n = 180	0.65 ± 9.9	−2.62 ± 8.8	−1.77 ± 7.4	−2.46 ± 60.5	−28.15 ± 56	7.79 ± 48.2
rs5882 GG/rs708272 AG	Yes, n = 10	0 ± 6.8	-	1 ± 2.2	43.17 ± 96	-	−1.20 ± 2.7
	No, n = 177	0.82 ± 10.1	-	−2 ± 7.6	−5.36 ± 56.7	-	8.75 ± 50.2
rs5882 GG/rs708272 GG	Yes, n = 5	−7.27 ± 12.3	−18 ± 0	−	−11.08 ± 73.3	−70 ± 0^v^	−
	No, n = 182	1.12 ± 9.8	−0.62 ± 7.9	−1.69 ± 7.3	−2.67 ± 59.2	−25.08 ± 54.6^v^	7.73 ± 47.7^v^

* General linear model adjusted for sex, baseline age, and body mass index variations at one year. SD: standard deviation. CETP: Cholesteryl ester transfer protein a Simvastatin versus no statin, significant P = 0.048 (corrected for false discovery rates). b Simvastatin versus no statin, significant P = 0.007 (corrected for false discovery rates). c Atorvastatin versus no statin, significant, P = 0.025 (corrected for false discovery rates). d Simvastatin versus no statin, significant P = 0.045 (corrected for false discovery rates). e Atorvastatin versus no statin, significant, P = 0.032 (corrected for false discovery rates). f Simvastatin versus no statin, significant, P = 0.018 (corrected for false discovery rates). g atorvastatin vs. no statin; P = 0.011 (corrected for false discovery rates). h simvastatin vs. no statin, P = 0.004 (corrected for false discovery rates). i Simvastatin versus no statin, P = 0.036 (corrected for false discovery rates). j Atorvastatin versus no statin; uncorrected P = 0.049. k Simvastatin versus no statin, significant, P = 0.007 (corrected for false discovery rates). l Haplotype effect, atorvastatin versus no statin, significant P = 0.043 (corrected for false discovery rates). m simvastatin versus no statin; P = 0.027 (corrected for false discovery rates). n Simvastatin versus no statin, P = 0.011 (corrected for false discovery rates). o Haplotype effect, atorvastatin versus no statin, significant P = 0.041 (corrected for false discovery rates). p Simvastatin versus no statin, P = 0.029 (corrected for false discovery rates). q Simvastatin versus no statin, significant P = 0.018 (corrected for false discovery rates). r Simvastatin versus no statin, P = 0.023 (corrected for false discovery rates). s Haplotype effect, atorvastatin versus no statin, P = 0.011 (corrected for false discovery rates). t Simvastatin versus no statin, P = 0.002 (corrected for false discovery rates). u Atorvastatin versus no statin, significant P = 0.039 (corrected for false discovery rates). v Atorvastatin versus no statin, significant P = 0.034 (corrected for false discovery rates).

Since no genotype variations were observed in rs3846662 (HMGCR), this gene could not be included in the pharmacogenetic analyses. In addition, considering the low variability of the genotypes of rs11669576 (LDLR8), which led to the underrepresentation of some LDLR haplotypes, only CETP haplotypes were considered in the analyses.

Likewise, the isolated results of rosuvastatin versus no statin or other statins (atorvastatin or simvastatin) were not calculated because only two patients used rosuvastatin.

## DISCUSSION

Overall, this study showed that lipophilic statin therapy was effective in reducing total cholesterol and LDL-cholesterol levels after one year, which is consistent with previous literature,^24^ because lipophilicity eases the distribution of statins into hepatocytes.^25^ Particularly, simvastatin was more effective in elevating HDL-cholesterol levels, while atorvastatin was more effective in reducing triglyceride levels. Notably, the only genetic variant that resulted in significant lipid profile variation, regardless of statin therapy, was the insertion allele of ACE, which cumulatively lowered total cholesterol and LDL cholesterol. The effect of the insertion allele of ACE on lowering serum levels of the angiotensin-converting enzyme could explain its protective effect on lipid profiles, considering that angiotensin II upregulates the enzyme 3-hydroxy-3-methylglutaryl CoA reductase.^14^

Additionally, APOE-ε4 carriers showed a better response to atorvastatin in terms of elevated HDL-cholesterol levels compared to APOE-ε4 non-carriers. While Chinese APOE-ε2 carriers seem to have healthier lipid profiles even longitudinally,^26^ a Brazilian study showed that APOE-ε4 alleles do not interfere with the pharmacological efficacy of simvastatin.^6^ Furthermore, most studies show that APOE-ε4 carriers have higher total cholesterol and LDL-cholesterol, which are modifiable by diets.^5^ In contrast, the evidence for HDL-cholesterol is inconsistent.^27^ Rising HDL-cholesterol levels are also associated with functional harm for APOE-ε4 non-carriers with Alzheimer’s disease, probably because of reduced lipid availability to protect neuronal membranes.^28^ Moreover, the prospective side effects of psychotropic drugs are more intense according to APOE-ε4 carrier status.^29^ Likewise, APOE-ε4 carrier status is a key element in the modulation of effects of cerebrovascular metabolism modulators.^30^ Thus, stratification of patient samples according to APOE-ε4 carrier status may be the best alternative.^31^

APOE-ε4 carriers with Alzheimer’s disease appear to have a distinct lipid profile that may lead to higher susceptibility to the disease,^27^ with increased intestinal cholesterol absorption^32^ and decreased plasma levels of the less lipidated apolipoprotein E4. In contrast, APOE-ε2 carriers have increased levels of the more lipidated apolipoprotein E2 which binds with lower affinity to the low-density lipoprotein receptor than the other isoforms.^13^ In addition, patients with Alzheimer’s disease have abnormal insulin metabolism associated with the apolipoprotein E,^33^ corroborated by reports of reduced hippocampal insulin-degrading enzyme (an amyloid-β-degrading metalloprotease) in APOE-ε4 carriers.^34^

In this study, simvastatin use resulted in cumulatively lower total cholesterol and LDL-cholesterol levels in carriers of the insertion allele of ACE, as well as lower triglyceride levels in carriers of the D/D genotype. Conversely, atorvastatin lowered triglyceride levels, mostly in carriers of the insertion allele of ACE. A previous study showed that carriers of the deletion allele had a better response to fluvastatin in terms of reductions in triglycerides and LDL-cholesterol.^25^ Although the insertion allele is associated with improved pharmacological protection against creatinine clearance variations,^8^ which allele would confer protective effects against the lipid-lowering effects of statins remains controversial, considering that both alleles can behave in the same way under different conditions.

Simvastatin use resulted in lower total cholesterol and LDL-cholesterol levels whereas atorvastatin use resulted in lower total cholesterol and triglyceride levels in carriers of the G allele of rs11669576 (LDLR8). However, no significant results were found for rs5930 (LDLR10), even though the A allele has been described as protective in terms of behavioral features^15^ and creatinine ­clearance variations.^8^ Moreover, presence of the G allele of rs11669576 or the G allele of rs5930 is associated with upregulation of an abnormal low-density lipoprotein receptor that inhibits the internalization of the apolipoprotein E (thus reducing its transfer to lipoproteins for clearance), resulting in increased synthesis of low-density lipoproteins and reduced storage of intracellular cholesterol while precluding recycling of the low-density lipoprotein receptor.^13^ Furthermore, no significant differences were observed in rs2695121 (NR1H2). Although the T allele is associated with a higher behavioral burden^15^ and lower blood pressure, conflicting results have been reported regarding its association with the metabolic syndrome.^8^

When using atorvastatin, carriers of the GG genotype of rs5882 (CETP) had lower LDL cholesterol and triglyceride levels, whereas carriers of the A allele of rs708272 (CETP) had higher increases in HDL cholesterol levels. Conversely, carriers of the GG genotype of rs708272 (CETP) showed lower HDL-cholesterol levels when using atorvastatin. The G allele of rs5882 (CETP) is associated with lower serum cholesteryl ester transfer protein levels and greater white matter integrity in young adults, as well as preserved cognitive function in longevity,^15^ while the A allele of rs708272 (CETP) is associated with lower serum cholesteryl ester transfer protein activity and lower coronary heart disease risk.^35^ Although one meta-analysis showed no associations between rs708272 (CETP) and the lipid-lowering effects of statin therapy,^36^ rs708272 (CETP) may explain up to 10% of the variation in HDL-cholesterol levels,^37^ with rs5882 (CETP) playing a similar role.^38^

Carriers of CETP haplotypes that included the A allele of rs5882 and the A allele of rs708272 benefited from statins, which lowered total cholesterol and raised HDL-cholesterol. Specifically, they benefitted from lower triglyceride levels with atorvastatin. However, the most pronounced effects of atorvastatin on lowering total cholesterol and LDL cholesterol were observed in carriers of rs5882 GG/rs708272 GG. The G alleles of both single nucleotide polymorphisms are associated with fewer frontal behaviors,^15^ suggesting that CETP haplotypes associated with neuroprotection may also confer lower total cholesterol levels with atorvastatin therapy.

Nonetheless, this study has some limitations. First, it is limited by its convenience sample with no measurements of dietary patterns that could result in different lipid profiles according to specific genetic variants.^39^ Second, although our longitudinal reading could identify significant differences for several associations, despite the absence of variability in rs3846662 (HMGCR) genotypes, the modest sample size is an inherent problem in most single-center studies. Despite this, almost three-quarters of our patients had dyslipidemia, confirming the burden of this vascular risk factor on patients with Alzheimer’s disease. Third, interpretation of the results is limited by the lack of randomization of the sample or stratification according to environmental factors, as well as the absence of measures of the proteins that are translated by the studied genes. However, whether statins would be more efficacious at the start of lipid-lowering therapy is unknown, since many patients were already under treatment when they were included in the study, which may have resulted in better lipid outcomes.^40^ Finally, physical activity could also be a confounding factor for our results; however, most patients were sedentary, thus, the effects of exercise were probably minimal.

## CONCLUSION

Our study showed that the genetic determinants of lipid profiles affected the individual variability in the response to statins in patients with Alzheimer’s disease, confirming the high heritability of lipid profile variations. Our findings may provide useful information for risk prediction and interventions in lipid disorders. Future genome-wide studies with larger sample sizes should address the effects of a greater variety of statins on lipid profile variations in patients of diverse age ranges.
